# A novel two‐hit murine model of inhaled occupational exposure‐induced lung disease

**DOI:** 10.14814/phy2.71019

**Published:** 2026-07-11

**Authors:** Melea Barahona, Ashley DeBie, Logan S. Dean, Bethany Klemp, Kaylee Jones, Emmanuel O. Oyewole, Mäelis Wahl, Morgan Pauly, Casey McDermott, Francisco J. Salguero, G. Brooke Anderson, Marcela Henao‐Tamayo, Tara M. Nordgren

**Affiliations:** ^1^ Cell and Molecular Biology Program Colorado State University Fort Collins Colorado USA; ^2^ Department of Environmental and Radiological Health Sciences Colorado State University Fort Collins Colorado USA; ^3^ Department of Microbiology, Immunology, and Pathology Colorado State University Fort Collins Colorado USA; ^4^ Department of Biochemistry and Molecular Biology Colorado State University Fort Collins Colorado USA; ^5^ One Health Institute, Universität Zürich Zurich Switzerland; ^6^ Institute of Evolutionary Medicine, Universität Zürich Zürich Switzerland; ^7^ Department of Biomedical Sciences Colorado State University Fort Collins Colorado USA; ^8^ Department of Pediatrics, College of Medicine University of Nebraska Medical Center Omaha Nebraska USA; ^9^ Toxicology Graduate Program Colorado State University Fort Collins Colorado USA; ^10^ United Kingdom Health Security Agency, UKHSA‐Porton Down Salisbury UK; ^11^ Faculty of Health and Medical Sciences University of Surrey Guildford UK

**Keywords:** environmental toxicants, inflammation, organic dust exposure (ODE), pulmonary fibrosis (PF)

## Abstract

The lungs are continuously exposed to environmental insults, rendering the lung epithelium vulnerable to damage and persistent inflammation. Such epithelial damage and prolonged inflammation are hallmark features of various lung diseases, including chronic obstructive pulmonary disease (COPD), pulmonary fibrosis (PF), and lung cancer. Organic dust exposure (ODE), prevalent among workers in livestock and agricultural sectors, is associated with significantly increased risk of these respiratory diseases, yet the underlying mechanisms relating these phenomena remain poorly understood. Here, we have generated a novel murine model of inhaled occupational exposure‐induced lung disease using a two‐hit approach combining exposure to inhaled organic dust and an established murine model of bleomycin‐induced PF to interrogate the signatures of lung damage onset and progression. Following the two‐hit treatment in C57Bl/6J mice, we identified a distinct immune cell profile in the bronchoalveolar lavage fluid (BALF), dominated by macrophages and lymphocytes. This response was accompanied by unique inflammatory and injury‐related histopathological changes in the lung that have not been observed in models using organic dust or bleomycin alone. These data, combined with additional physiological and cytokine findings, highlight the importance of applying multihit modeling in future work seeking to elucidate the cellular and molecular mechanisms associated with lung disease onset and progression.

## 1 INTRODUCTION

The lungs are continuously exposed to diverse environmental insults that increase the risk of chronic disease development. Occupationally relevant inhalable toxicants have been well recognized for their association with chronic lung inflammation and damage to the airway epithelium, with long‐term repetitive exposures leading to the development of chronic obstructive pulmonary disease (COPD) and pulmonary fibrosis (PF) (Feary et al., 2023; Kyung & Jeong, 2020; Spagnolo et al., 2023). COPD and PF are both debilitating and degenerative lung diseases, with COPD reigning as the fourth leading cause of death globally and PF being associated with a 2.5 to 3.5‐year median survival following diagnosis (World Health Organization, 2024; Kolb & Collard, 2014). Organic dust exposure (ODE) is a common occupational hazard for livestock and agricultural workers, with individuals working in large‐scale swine confinement facilities experiencing increased incidence of respiratory conditions such as hypersensitivity pneumonitis, asthma, bronchitis, COPD, and PF (Harting et al., 2012; May et al., 2012; Nordgren & Bailey, 2016; Poole et al., 2024; Spagnolo et al., 2023; Spurzem et al., 2002; Von Essen & Romberger, 2003). Treatment options for patients with COPD and PF remain limited, as current therapies primarily manage symptoms rather than targeting the underlying mechanisms driving progressive lung tissue remodeling and functional decline (Aribindi et al., 2024; Rodrigues et al., 2021). The development of robust murine models that simulate inhaled occupational exposure‐induced lung disease will aid in filling this gap and support future investigations seeking to reveal the cellular and molecular mechanisms associated with chronic inflammation and resolution responses.

Human lung diseases are rarely initiated by a singular injurious event or insult, but existing chronic murine models are commonly dependent on individual toxicant exposures, limiting the translational relevance of serial multihit pulmonary challenges. Previous studies from our group have demonstrated that repetitive organic dust inhalation induces persistent lung inflammatory pathology and significant tissue remodeling in mice that is consistent with fibrotic changes observed in agricultural workers (Dominguez et al., 2022; Poole et al., 2009; Warren et al., 2017). While this model recapitulates prominent fibrotic pathology in the lungs, the mechanisms that drive this process remain poorly understood (Dominguez et al., 2022). Conversely, the murine bleomycin (BLM) model of PF is commonly used to investigate the cellular and molecular mechanisms underlying fibrotic lung disease progression; however, this type of model rarely incorporates inhaled particulate exposures that humans encounter prior to developing clinical signs of disease (Gul et al., 2023; Ishida et al., 2023; Kadam & Schnitzer, 2024). To address these limitations, an exposome‐informed approach can be used to address repeated and heterogeneous inhalational exposures that collectively shape the inflammatory milieu over time (Akgün, 2025; Guillien et al., 2023). Thus, developing a novel murine model incorporating more than one lung injury‐inducing insult is key to uncover translationally relevant mechanistic underpinnings of disease (Lang & Hickman‐Davis, 2005).

In the present study, we introduce and characterize a novel two‐hit murine model of lung disease combining two well‐established models of inhaled occupational exposure to organic dusts and BLM‐induced PF. We demonstrate that the two‐hit model induces mild to moderate fibrotic changes within the lungs with distinct immunological features, setting it apart from the features associated with each individual model. Our findings suggest the utility of multihit models in elucidating unique cellular and molecular mechanisms of damage and repair in the context of exposure‐induced lung injury.

## 2 MATERIALS AND METHODS

### 2.1 Preparation of organic dust extract (DE)

Organic dust was collected, and dust extract (DE) was prepared as previously described (Nordgren et al., 2015; Romberger et al., 2002; Ulu et al., 2022). In brief, settled dust from swine confinement facilities located in the Midwestern United States was collected from surfaces at least 1 meter from the ground to represent the respirable portion. Dust was subsequently suspended in Hank's Buffered Saline Solution (HBSS) at 1 g of dust per 10 mL HBSS and incubated at room temperature for 1 h. The aqueous dust solution was centrifuged at 1188 g/2500 rpm (16.8 cm rotor) for 20 min at 4°C. The supernatant fraction was collected and subjected to an additional centrifugation using the parameters outlined previously, and the pellet was discarded. The final supernatant fraction from the second centrifugation step was collected and passed through a 0.22 μm syringe filter to produce 100% DE. The 100% DE was aliquoted and stored at −20°C prior to use. The prepared 100% DE is composed of gram‐positive bacterial components, endotoxins, and trace metals. A complete analysis of the DE can be found in the “Organic dust and ODE analysis” heading of the Methods section of the Online Repository at https://www.jacionline.org/article/S0091‐6749(08)00966‐4/fulltext#app‐1 (Poole et al., 2008, 2010).

### 2.2 Animal care and housing

All mouse studies were conducted according to protocols reviewed and approved by the Colorado State University (CSU) Institutional Animal Care and Use Committee (Protocol Number 2887). Male and female C57BL/6J (WT) mice (Jackson Labs) aged 7–14 weeks were group housed by sex in groups of 5 in purple individually ventilated cages on racks (Allentown, NexGen Mouse 500) at the CSU Painter Center and allowed free access to standard mouse chow (NIH Mouse and Rat Ration 5018, Cincinnati Lab Supply, Cat# 0050969) and water.

### 2.3 In vivo DE exposure model

An established in vivo murine model of intranasal (IN) inhaled repetitive exposure to DE was utilized (Poole et al., 2008, 2009; Warren et al., 2017). In brief, mice were lightly anesthetized with aerosolized isoflurane delivered using a small animal anesthesia box fitted to a SomnoSuite Small Animal Anesthesia System (Kent Scientific Corporation) prior to receiving intranasal instillation of either 50 μL sterile phosphate‐buffered saline (PBS) or 12.5% DE daily for 3 weeks (5 days/week), sacrificing 5 h post final instillation. The 12.5% DE concentration utilized in this study was previously determined to be both tolerable and optimal for experimental outcomes (Poole et al., 2009; Warren et al., 2017).

### 2.4 In vivo bleomycin (BLM) pulmonary fibrosis model

An established in vivo murine model of BLM administered via oropharyngeal aspiration (OA) was utilized (De Vooght et al., 2009; Egger et al., 2013). In brief, mice underwent light anesthesia with isoflurane as described in the section above. A dose of 0.5 mg/kg bleomycin for injection, USP (Meitheal Pharmaceuticals, Chicago, Illinois, NDC: 71288‐106‐10) or 0.9% sterile saline vehicle was administered via OA in a volume equivalent to 1 μL/gram of mouse for 6 consecutive days, followed by 21 days of monitoring to allow for fibrosis development prior to sacrifice.

### 2.5 In vivo two‐hit model

**FIGURE 1:**
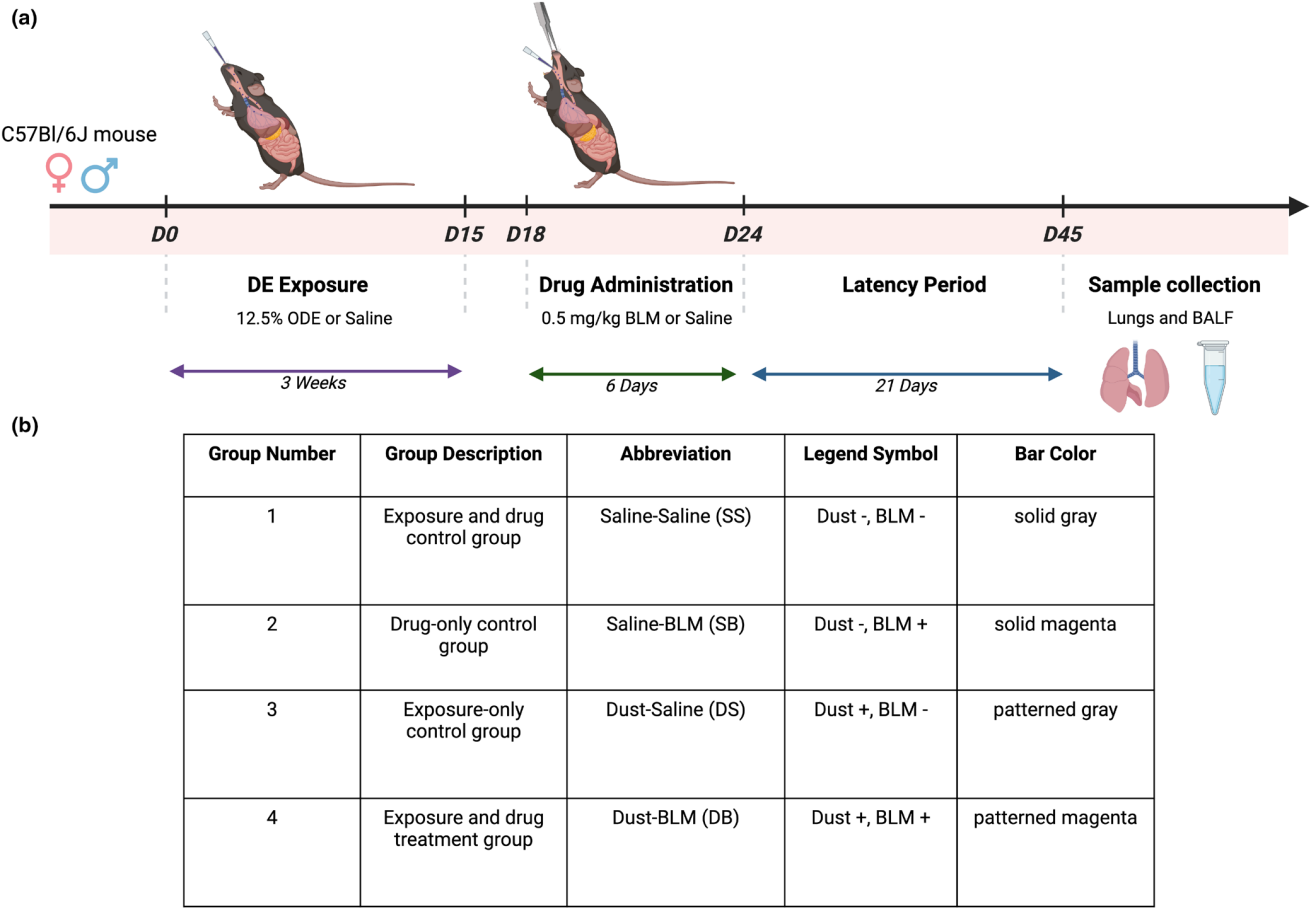
Two‐hit occupational exposure‐induced lung disease experimental design. (a) Timeline of DE exposure and BLM administration. (b) Group designations and definitions. Created in BioRender. Henao‐Tamayo, M. (2026) https://BioRender.com/k7ow3c1.

The two‐hit in vivo murine model incorporated the DE exposure regimen outlined above, followed by 2 days of rest prior to BLM administration following the OA method stated above (Figure 1a). The treatment groups are described in the table unless otherwise stated (Figure 1b).

### 2.6 Pulse ox, RR, weight collection

Within the 21 days following the final BLM administration, mice were periodically weighed and lightly anesthetized as described above for respiratory rate (RR) and peripheral oxygen saturation (SpO_2_) data collection. RR was collected by manually counting breaths taken in 10 s and subsequently multiplied by 6 to obtain breaths per minute (brpm). SpO_2_ was collected using the MouseSTAT® attachment outfitted to work with the SomnoSuite Small Animal Anesthesia System (Kent Scientific).

### 2.7 Animal euthanasia and sample collection

Following the completion of each model timeline, mice were euthanized according to guidelines set forth by the American Veterinary Medical Association, whereby mice were subjected to isoflurane overdose followed by cervical dislocation (https://www.avma.org/resources‐tools/avma‐policies/avma‐guidelines‐euthanasia‐animals). Bronchoalveolar lavage fluid (BALF) and lung tissue samples were collected as previously described (Nordgren et al., 2015; Threatt et al., 2024; Ulu et al., 2022). In brief, BALF was obtained using 3 × 1 mL aliquots of ice‐cold PBS (HyClone Laboratories, SH3025602) to lavage the lungs. The supernatant fraction from the first lavage was aliquoted and stored at −80°C for cytokine analyses using enzyme‐linked immunosorbent assays (ELISA). The pellets from each lavage were combined, underwent red blood cell lysis (Gibco, A1049201), and were used for total and differential cell count analyses. The left lung lobes were tied off, removed, and stored in RNAlater (Invitrogen, AM7020) at −80°C until use. The right lung lobes were inflated with 10% neutral‐buffered formalin (NBF) (Cancer Diagnostics Inc., FX1002) under 20 cm of pressure and stored in 10% NBF for at least 24 h prior to submission to the CSU Veterinary Diagnostic Laboratory Experimental Pathology Facility (EPF) for paraffin embedding and sectioning.

### 2.8 Lung histopathology

Paraffin‐embedded formalin‐fixed right upper and lower lung lobes were mounted together and sliced as described above into 5 μm tissue sections placed on slides. Single slides containing both lung lobes from each mouse were deparaffinized and rehydrated using a graded ethanol approach for subsequent staining with hematoxylin and eosin (H&E). An additional slide from each mouse was stained with Masson's Trichrome Stain by the EPF. Stained slides were whole‐image montage scanned using 20× magnification on an Olympus VS120 Scanning Microscope located in the EPF. The scanR software (Olympus/Evident Scientific; software version 4.3) associated with the VS120 was utilized for image acquisition and automatically stitched each section on a single slide into nonoverlapping fields of view across the entire tissue area for analysis. The number of fields per section was determined by the scanR software and was consistent across samples. All fields within each section were included in the analysis; no manual selection of fields was performed. Scanned H&E‐stained slides were blinded and scored by a certified pathologist for inflammatory pathology using previously established scoring criteria (Ulu et al., 2022; Warren et al., 2017). The four scoring parameters were based on the amount of immune cell aggregates, the degree of perivascular and peribronchiolar inflammation, cellularity of the alveolar spaces, and goblet cell metaplasia within the bronchial spaces, similar to previously described (Poole et al., 2009; Ulu et al., 2022; Warren et al., 2017). Scanned Masson's Trichrome stained slides were blinded and scored by a certified pathologist for fibrotic pathology using the previously established modified Ashcroft scale guidelines published by Hubner, et al. (Ashcroft et al., 1988; Hübner et al., 2008).

### 2.9 Cytokine quantification

The supernatant fraction from the first lavage of BALF was collected and stored as previously described above. Levels of interleukin‐6 (IL‐6), IL‐10, natural and recombinant transforming growth factor‐ β1 (TGF‐β1), and IL‐1β in BALF were assessed using R&D Systems DuoSet ELISA kits; mouse IL‐6 (Mouse IL‐6 DuoSet ELISA, DY406), IL‐10 (Mouse IL‐10 DuoSet ELISA, DY417), TGF‐β (Mouse TGF‐β1 DuoSet ELISA, DY1679), and IL‐1β (Mouse IL‐1 beta/IL‐1F2 DuoSet ELISA, DY401) were performed in accordance with manufacturer instructions. Left lung lobes were collected and stored as previously described above prior to homogenization. Homogenization of lung tissues was performed as previously described (Threatt et al., 2024). In brief, left lung lobes were homogenized using a Bead Mill 24 bead homogenizer in lysis buffer with PBS, 1X RIPA lysis buffer (Thermo Scientific, Cat#: J62524.AE), and 1X proteinase inhibitor cocktail (Thermo Scientific, Cat#: 1861279) using a ratio of 30 mg tissue to 1 mL buffer per individual sample, followed by a 30 min incubation on ice with agitation every 10 min. Lung homogenate aliquots were then stored at −80°C until use. Levels of collagen I, collagen III, and vimentin were assessed using the following ELISA kits: Mouse Collagen Type I ELISA Kit (Biotechne/Novus Biologicals, Cat#: NBP2‐75822), Mouse Collagen III ELISA Kit (Antibodies.com, Cat#: A76352), Mouse Vimentin ELISA Kit (Antibodies.com, Cat#: A312354). Upon assay completion, the plates were read using a Biotek Synergy HT Microplate Plate Reader located in the Colorado State University NTM Center. Protein concentration values were calculated according to the plate standards via a four‐parameter log–log fit model using GainData® (Arigo Biolaboratories, https://www.arigobio.com/elisa‐analysis).

### 2.10 Staining and analysis of differential cell counts from BALF

BALF cell pellets were resuspended and analyzed for total and differential cell counts using previously established methods (Nordgren et al., 2015; Threatt et al., 2024; Ulu et al., 2022). In brief, total cell counts were obtained using a Countess 3 FL automatic cell counter. These counts were then used to dilute cell suspensions with PBS to a final concentration of 1 × 10^6^ cells/mL prior to extracting 200 μL of this final cell suspension to add into a Thermo Cytospin 4 cytocentrifuge set to 600 rpm for 5 min. Upon completion of the centrifugation cycle, slides were allowed to dry overnight prior to staining with a Volu‐Sol dip‐stain kit (Volusol, VDS‐100). Stained slides were then imaged using 20× magnification on an Olympus VS120 Scanning Microscope located in the EPF and analyzed using standard counting methodology using QuPath image analysis software (QuPath‐0.5.1‐arm64) (Bankhead et al., 2017).

### 2.11 Statistical analysis

Differential cell counts and ELISA data were statistically and graphically analyzed using GraphPad Prism 10 (v 10.4.2 (534)). Changes in weight, RR, and SpO_2_ over time were statistically and graphically analyzed using R (v 4.5.2) and the following packages: ARTools package (v 0.11.2) for performing nonnormally distributed repeated measures ANOVA and associated contrasts of the ANOVA output, lme4 package (v 1.1–38) for performing repeated measures ANOVA on normally distributed data, and emmeans package (v 2.0.1) for multiple comparisons of the normally distributed ANOVA output. Data represented in bar graphs are depicted as the mean +/− the standard error of the mean (SEM). Data represented in line graphs are depicted as a single point for the mean of the replicates +/− standard deviation (SD). For determination of significance between two or more groups within each experiment, two‐ and three‐way ANOVA methods were used with Tukey's post‐hoc comparisons to account for multiple comparisons. If data were not normally distributed, nonparametric ANOVA methods were used. For Cytospin experiments with only two groups, the unpaired Student's *t*‐test was used on normally distributed data, and the Mann–Whitney rank test was used for nonnormally distributed data. All ELISA and differential cell count data underwent outlier analysis using the ROUT method with *Q* = 1% to exclude statistical outliers. Some of the raw protein levels from the ELISA data registered above the limit of detection (LOD). Acknowledging that a value cannot reliably be assigned to levels that fall above the LOD for the assay, these data could not be analyzed as continuous outcomes. Therefore, an alternative approach was taken where the data were categorized into five categories—Below LOD, Low, Mid, High, and Above LOD—and then subjected to statistical testing to assess the association between experimental group and categorized protein abundance. As such, we used the Fisher's Exact Test method for small sample sizes to determine the significance of the association. The results of this analysis are represented graphically as the normalized percentage of samples falling within each detection category (Below LOD, Low, Mid, High, Above LOD) in a contingency table generated by GraphPad Prism 10. Throughout, we used a significance level (*α*) of 0.05. Associated exact *p* values are annotated in each graph.

## 3 RESULTS

### 3.1 The two‐hit model exposure and treatment regimen does not adversely affect animal weight or lung physiological responses

**FIGURE 2:**
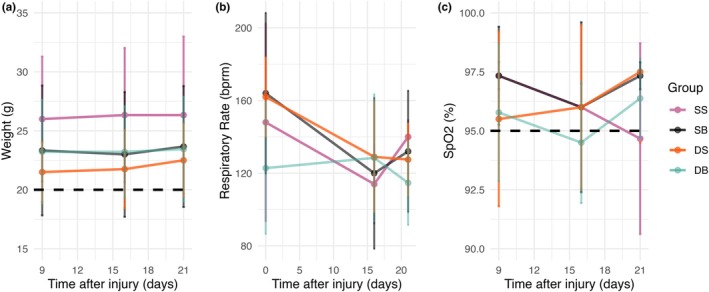
Physiological responses to the two‐hit exposure model in WT mice. (a) Body weight, (b) respiratory rate (RR), and (c) peripheral oxygen saturation (SpO2) were measured after treatment. The two‐hit model consisted of repeated DE administration IN for 5 days/week for a total of 3 weeks followed by 6 days of OA bleomycin (BLM) administration with 21 days of lung disease development allotted prior to sacrifice. The groups are designated in the following colors: SS in magenta, SB in black, DS in orange, and DB in teal. Data points are presented as mean +/− SD of biological replicates in each group. Statistical significance was assessed using repeated measures ANOVA with post hoc Tukey's test. No statistical significance was observed following analysis. SS, *N* = 3; SB, *N* = 3; DS, *N* = 4; DB, *N* = 9.

We initially sought to assess animal health longitudinally across the two‐hit timeline, using weight, respiratory rate (RR), and peripheral oxygen saturation (SpO_2_) as indicators of animal health. We did not observe any statistically significant changes or trends in weight loss or gain following the two‐hit regimen during the latency period from days 9 to 21 (Figure 2a). Once we established that this model had a 0% mortality rate and no significant effect on animal weight, we assessed lung physiological responses to the two‐hit model. As expected, we saw no statistically significant changes or trends in RR or SpO_2_ between experimental groups over the course of the 21‐day latency period (Figure 2b,c).

### 3.2 Observed lung tissue fibrosis is mild and driven by BLM exposure

**FIGURE 3:**
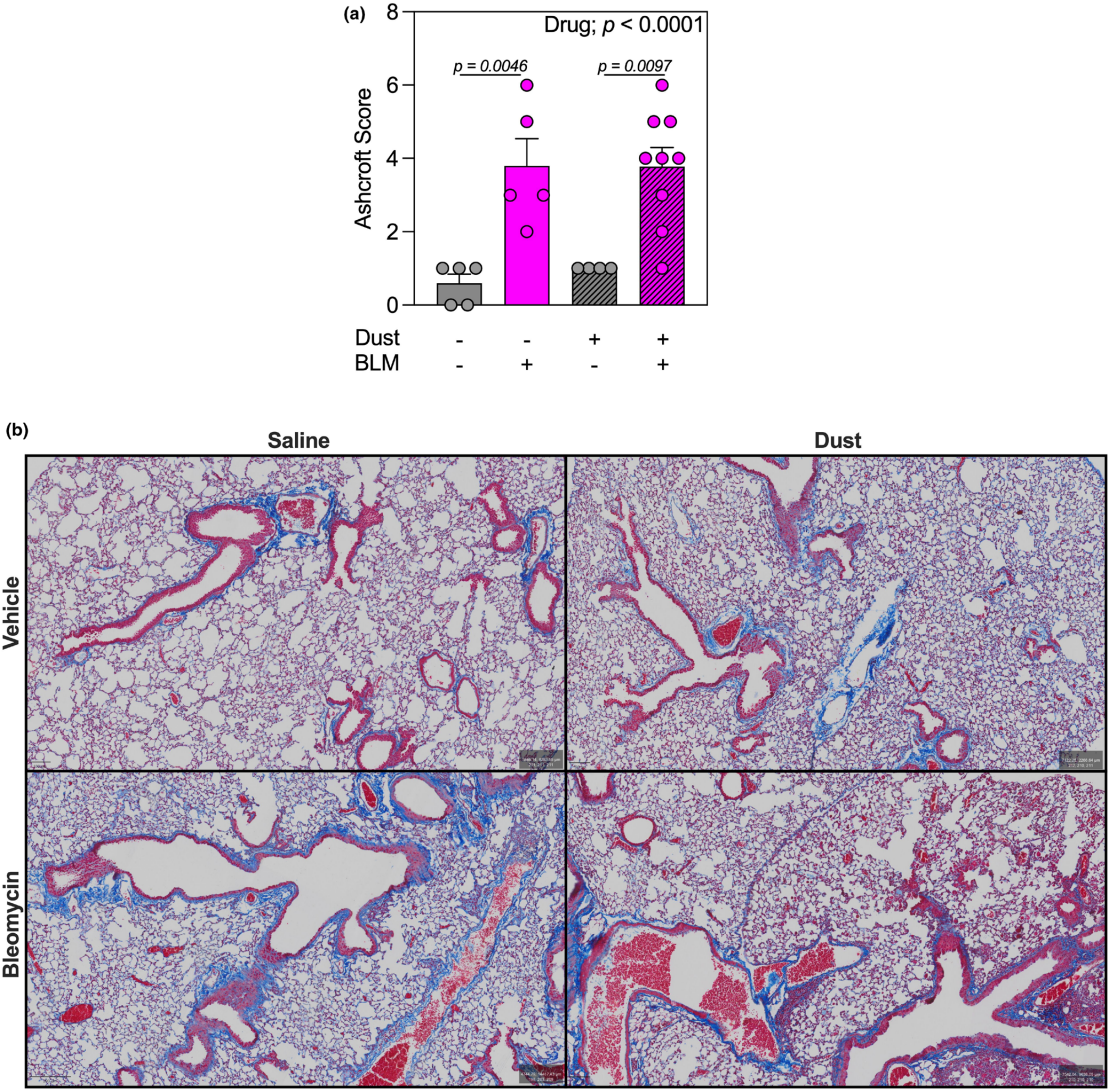
Lung fibrosis histopathology and scoring following the two‐hit exposure model in WT mice. (a) Ashcroft score using sectioned left lung lobes after treatment. The lungs were stained with Masson's Trichrome stain to visualize (b) fibrotic changes (imaged at 20×). Scale bars were set at 100 μm for examination of tissue remodeling. Main effects between three or more groups were determined by 2‐way ANOVA. Error bars are depicted as SEM. Statistical significance between multiple comparisons from Tukey's post‐hoc analysis. Each point on the bar graph represents an individual mouse.

Lung tissues stained with Masson's Trichrome stain were blindly scored for fibrotic change by a board‐certified pathologist using the modified Ashcroft scale guidelines published by Hubner, et al. (Ashcroft et al., 1988; Hübner et al., 2008). As expected, administration of BLM (Drug) had a significant main effect on fibrotic change observed in lung tissues (*p* < 0.0001) (Figure 3a,b). Multiple comparisons between experimental groups yielded significant differences between the SS group and SB group (*p* < 0.01) as well as the DS group and DB group (*p* < 0.01) (Figure 3a,b).

### 3.3 Organic dust exposure drives distinct lung inflammatory pathology in two‐hit model mice

**FIGURE 4:**
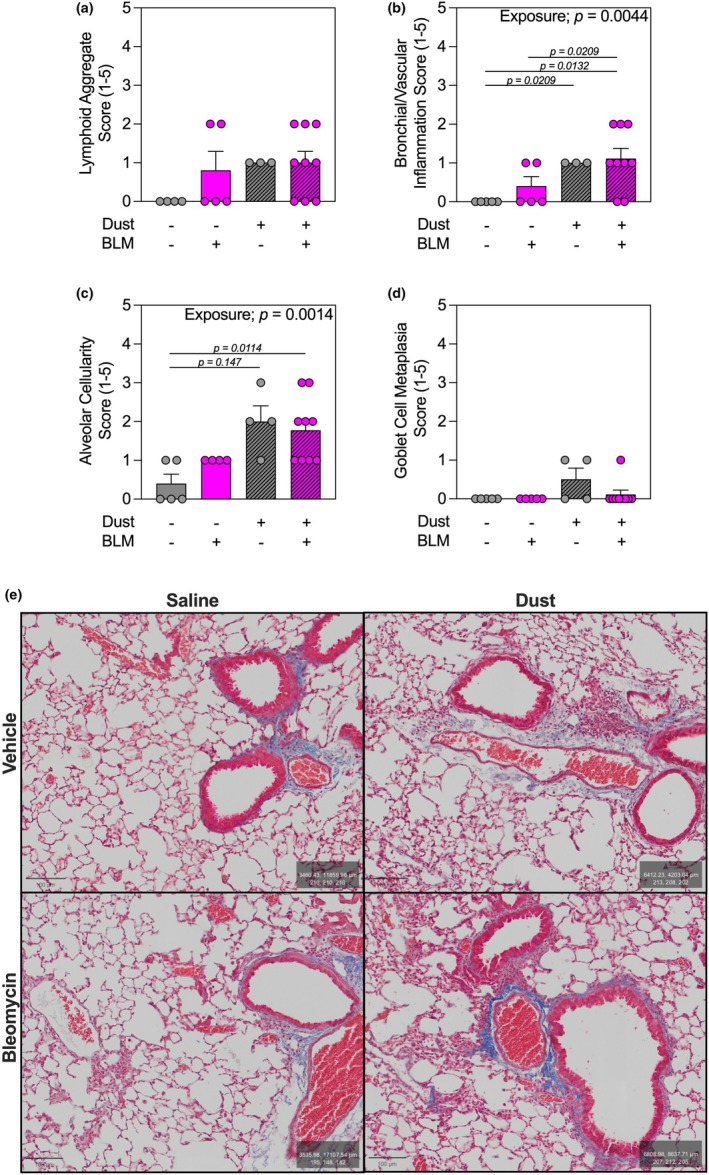
Lung inflammation histopathology and scoring following the two‐hit exposure model in WT mice. (a) Lymphoid aggregates, (b) bronchial/vascular inflammation, (c) alveolar cellularity, (d) and goblet cell metaplasia were scored blindly using sectioned left lung lobes following the two‐hit regimen. The lungs were stained with Masson's Trichrome stain to visualize (e) inflammatory pathology (imaged at 20×). Scale bars were set at 100 μm for examination of inflammation. Main effects between three or more groups were determined by 2‐way ANOVA. Error bars are depicted as SEM. Statistical significance between multiple comparisons was determined from Tukey's post‐hoc analysis. Each point on the bar graphs represents an individual mouse.

Lung tissues stained with H&E were blindly scored for pathological changes associated with inflammation by a board‐certified pathologist using a previously established scoring criteria that assess lymphoid aggregate formation, bronchial and vascular inflammation, alveolar cellularity, and goblet cell metaplasia (Poole et al., 2009; Ulu et al., 2022; Warren et al., 2017). We observed no statistically significant differences in lymphoid aggregate abundance in the lung tissues of any of the experimental groups (Figure 4a). We did, however, observe significant differences in bronchial and vascular inflammation in the groups exposed to organic dust. Multiple comparisons of the experimental groups showed significant differences between the DS group and SS group (*p* < 0.05), DB group and SS group (*p* < 0.05), and DB group and SB group (*p* < 0.05) (Figure 4b). Additionally, we observed significant differences in alveolar cellularity between experimental groups with exposure to DE. Multiple comparisons of the experimental groups showed significant differences in this metric between the DS group and DB group compared to the SS group (*p* < 0.05, *p* < 0.05), respectively (Figure 4c). We did not observe significant changes in goblet cell metaplasia scores between experimental groups (Figure 4d). Representative images of lung structures from each experimental group show that groups that received DE have more inflammatory pathological features than their saline receiving counterparts (Figure 4e).

### 3.4 Neither structural protein expression linked to lung tissue remodeling nor inflammatory cytokine markers are significantly influenced by the two‐hit model regimen

**FIGURE 5:**
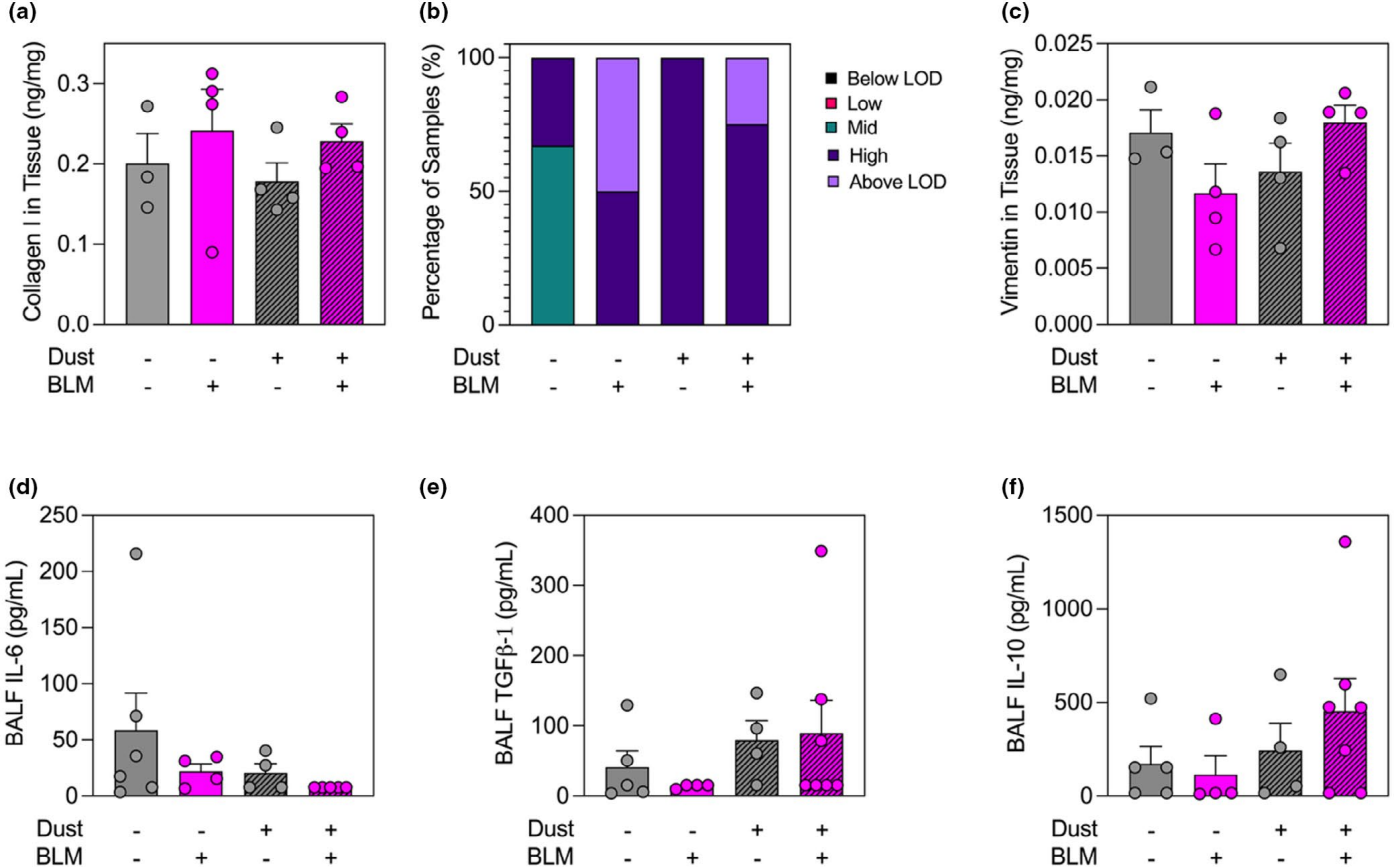
Effects of the two‐hit model on structural protein markers of tissue remodeling in lung tissue homogenates and cytokines associated with inflammatory processes in BALF from WT mice. (a) Collagen I protein levels in tissue, (b) trends of collagen III protein levels in tissue (all groups had an *N* = 3–4), and (c) vimentin protein levels in tissue. (d) IL‐6 cytokine levels in BALF, (e) total TGFβ‐1 cytokine levels in BALF, (f) IL‐10 cytokine levels in BALF. Main effects and multiple comparisons were assessed using 2‐way ANOVA, but no statistical significance was observed. For collagen III, the Fisher's Exact test was performed. Error bars are depicted as SEM. Each point on the bar graphs represents an individual mouse.

After assessing the resultant fibrotic and inflammatory pathological changes in lung tissue following the two‐hit model regimen, we were interested in characterizing changes in the abundance of specific structural proteins. We observed no statistically significant changes in collagen I following the two‐hit model regimen, indicating that there were no significant changes in mature collagen content within the lung (Figure 5a). We then sought to understand if active tissue remodeling was occurring in our model. We observed samples with collagen III protein levels that fell above the limit of detection (LOD). We thus used a categorical approach to analyze the collagen III data by way of Fisher's Exact Test to determine the significance of the association between experimental group and protein detection levels. The Fisher's Exact Test did not detect a statistically significant association between experimental groups (SS, SB, DS, and DB) and protein detection level (Below LOD, Low, Mid, High, Above LOD) (*p* = 0.1089). We observed that only the SB and DB groups had a percentage of samples that fell above LOD for collagen III, indicative of active tissue remodeling occurring following BLM drug treatment. Interestingly, we observed that 100% of DS sample values were within the High range of the LOD for collagen III, indicating that lung damage from the DE alone initiated active collagen deposition (Figure 5b). We then investigated vimentin protein levels to identify if there is an increase in fibroblast and myofibroblast activity following the two‐hit regimen. While we anticipated an increase in vimentin protein levels in the DB and SB groups, we observed no statistically significant changes in vimentin levels between groups (Figure 5c). However, two‐way ANOVA analysis identified the potential for the interaction between Exposure X Drug to be a main effect (*p* = 0.0555) (Figure 5c). Next, we investigated levels of cytokine markers of inflammatory processes present in the BALF samples. We observed no statistically significant differences in levels of IL‐6, total TGFβ‐1, or IL‐10 in the BALF following the two‐hit regimen (Figure 5d–f).

### 3.5 Lung immune cell populations present following the two‐hit model regimen are distinct from the populations observed in individual models

**FIGURE 6:**
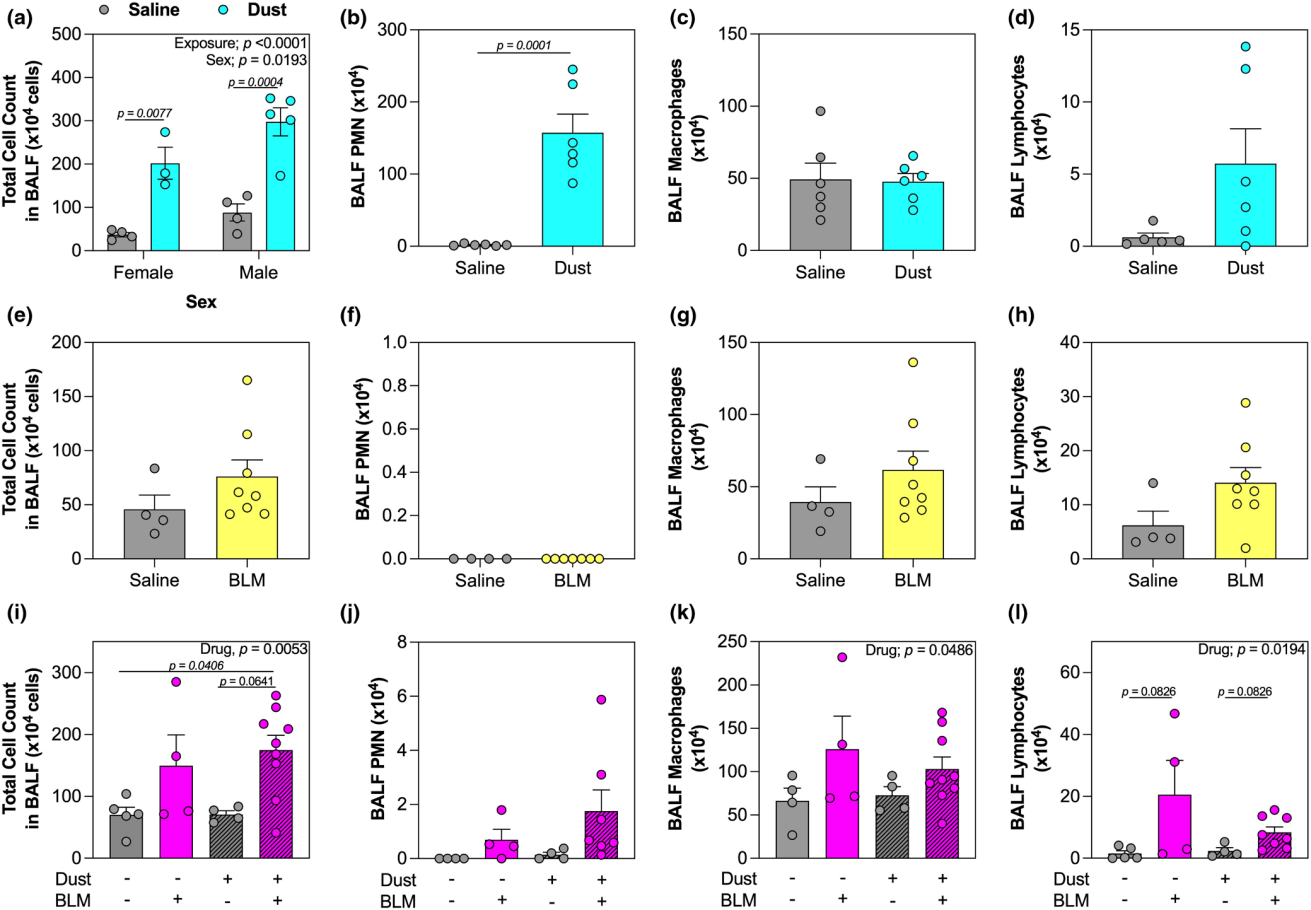
Modulation in immune cell infiltration and populations within the lung following DE only, BLM only, and the two‐hit model in WT mice. Following 3 weeks of 12.5% DE exposure, WT mice were euthanized 5 h after the final exposure and the collected BALF was assessed for total (a) immune cell counts, (b) neutrophils, (c) macrophages, and (d) lymphocytes. Following 6 consecutive days of 0.5 mg/kg BLM administration and 21 days allotted for lung disease development, WT mice were euthanized and the collected BALF was assessed for total (e) immune cell counts, (f) neutrophils, (g) macrophages, and (h) lymphocytes. Following completion of the two‐hit model timeline scheme of repeated DE administration IN for 5 days/week for a total of 3 weeks followed by 6 days of OA bleomycin (BLM) administration with 21 days of lung disease development allotted prior to sacrifice, WT mice were euthanized and the collected BALF was assessed for total (i) immune cell counts, (j) neutrophils, (k) macrophages, and (l) lymphocytes. Main effects between three or more groups were determined by 2‐way ANOVA and are indicated on each figure. The unpaired student's *t*‐test for normally distributed data and the Mann–Whitney rank test for nonnormally distributed data were used to determine significance between two groups and statistical significance. Error bars are depicted as SEM. Statistical significance between multiple comparisons from Tukey's post‐hoc analysis. Each point on the graphs represents an individual mouse.

Next, we characterized the total and differential immune cell counts in the BALF of animals from each individual model for comparison to the two‐hit model schema. We observed a previously described sex‐specific increase in total immune cell counts in the repetitive DE exposure‐only model (Females; *p* < 0.01, Males; *p <* 0.001), with main effects of both Exposure (*p* < 0.0001) and Sex (*p* = 0.0193) following two‐way ANOVA analysis (Figure 6a) (Ulu et al., 2022). For the differential immune cell counts, we did not observe sex‐specific differences. We observed a statistically significant increase in neutrophils following repetitive DE inhalation, consistent with previously published literature using this model (*p* < 0.001) (Figure 6b) (Poole et al., 2009; Ulu et al., 2022; Warren et al., 2017). We did not observe significant differences in macrophage abundance between groups (Figure 6c). In contrast, lymphocyte frequencies appear to differ between groups graphically, but these differences did not reach statistical significance (*p* = 0.0888) (Figure 6d). In the BLM‐only model, we did not observe a statistically significant increase in total immune cell counts or differential cell counts of neutrophils, macrophages, or lymphocytes (Figure 6e–h). We then characterized the total immune cell counts and differential immune cell counts from the two‐hit model to assess for alterations in immune cell populations and abundances. Here, we observed a significant Drug main effect (*p* = 0.0053) as well as significant increases in total immune cell counts between the SS group and the DB group (*p <* 0.05), with a trend observed between the DS group and the DB group (*p* = 0.0641) (Figure 6i). Increases in neutrophils following the two‐hit exposure between the DB and SB groups did not reach statistical significance in post‐hoc analyses, despite observing a potential Drug main effect (*p* = 0.0717). We did not observe any differences in macrophage counts between experimental groups, but we did identify a significant Drug main effect on the abundance of macrophages (*p* = 0.0486) (Figure 6k). Additionally, we did not observe significant changes in lymphocyte counts between experimental groups, but we did observe increased trends between the SS and SB groups (*p* = 0.0826) and the DS and DB groups (*p* = 0.0826), respectively. However, we did observe a significant Drug main effect (*p* = 0.0194) on lymphocyte counts in the two‐hit model (Figure 6l).

## 4 DISCUSSION

In the present study, we developed and characterized a novel two‐hit murine model of inhaled occupational exposure‐induced lung disease by combining established models of inhaled organic dust and BLM‐induced PF. Previous models have been utilized to mimic lung injury in response to a single insult, which fail to recapitulate the multifactorial nature of exposure‐induced human disease. By integrating two insults, organic dust and BLM, our novel murine model serves as a practical step towards bringing the epidemiological concept of the exposome into a controlled experimental setting, allowing us to better capture a more translationally relevant view of immune‐driven injury and repair responses (Akgün, 2025; Guillien et al., 2023; Lang & Hickman‐Davis, 2005). With this model, we aimed to use an exposome‐informed approach to uncover mechanistic differences in immune modulation that are not apparent in single‐injury models. Our results reveal important insights into the lung damage and repair responses we may expect to see following serial inhaled toxicant exposure.

We used a multiparametric approach to assess lung disease onset and severity resulting from our model, including measures of weight, RR, and SpO_2_ to track animal health and lung physiological effects. We did not observe statistically significant changes in animal weight, RR, or SpO_2_ in the 21 days following the final BLM administration. With no need to sacrifice animals due to significant weight loss prior to the final endpoint, we were able to collect robust lung physiological data across the duration of the study. Weight loss caused by oropharyngeal administration of BLM has been inconsistently reported in the literature. A 2013 study by Egger, et al. using 0.25 mg/kg BLM over a six‐day administration regimen reported 5 out of 8 animals needed to be sacrificed prior to the study conclusion due to considerable weight loss (>20%) (Egger et al., 2013). Additionally, in a 2018 study by Barbayianni, et al., authors found that using a higher dosage of BLM at 0.8 U/kg in a single administration resulted in no animal mortality or considerable weight loss. In alignment with the study done by Barbayianni, et al., we report similar findings related to animal survival and animal weight (Barbayianni et al., 2018). We interpret this physiological data to suggest that subsequent observed cellular and molecular changes are not due to the potential toxicity of the model but are likely specific to localized lung injury and repair responses. The tolerance to the exposure regimen aids in supporting its future use as a candidate for modeling sub‐chronic lung tissue remodeling more reflective of real‐world occupational exposure scenarios than single‐hit models alone. This aspect of our model is ideal for mimicking early stages of fibrotic development that may precede the onset of diseases like COPD and PF in farming communities, which often develop silently over time.

Despite a lack of notable physiological changes from the two‐hit model, we observed histopathological changes indicative of persistent inflammation and fibrotic development in our DB two‐hit experimental group. Mild to moderate fibrotic development has been previously described in individual models of chronic repetitive DE exposure using a 6‐month regimen or BLM‐induced pulmonary fibrosis via oropharyngeal aspiration (Barbayianni et al., 2018; Dominguez et al., 2022). It was unclear how using both exposures in this two‐hit model would affect inflammatory and fibrotic histopathological outcomes. We observed fibrotic tissue changes within the lungs of two‐hit animals that were consistent with BLM‐only animals. These observations then led us to investigate the inflammatory profile of the lung tissue. We found that, indeed, DB two‐hit experimental animals and DS exposure‐only control animals had higher pulmonary inflammatory scores for both bronchial/vascular inflammation and alveolar cellularity. These findings are consistent with previously reported data indicating that repetitive organic dust exposure induces long‐term inflammation that is slow to resolve over time (Warren et al., 2017). Notably, the inflammatory scores of the DB two‐hit experimental animals and DS exposure‐only control animals were significantly different from those of the SB drug‐only control animals, as this group had low inflammatory scores in all categories. We attribute this difference in inflammatory histopathological profile to the nature of the inflammatory response elicited by organic dust versus a drug like BLM. It is noticeable from these data that our two‐hit model initiates unique histopathological features within the lungs following repetitive exposure to heterogenous insults. Future investigations into physiological parameters indicative of fibrosis, such as assessment of lung compliance through pressure‐volume curve (P‐V loop) analysis, would provide additional insight and more robustly connect functional pulmonary outcomes with the histopathological observations.

Following histopathological scoring for disease and assessment of inflammation, we further characterized the lung damage from the model using canonical markers of tissue remodeling (collagen I, collagen III, vimentin). We expected to see changes in structural protein levels consistent with early fibrotic tissue remodeling resulting from the lung damage induced by the two‐hit model. While changes in structural proteins' abundance have not been demonstrated in lung tissue following the established 3‐week model of organic dust exposure, it has been shown that these proteins are modulated in models of chronic repetitive DE exposure using a 6 month regimen, as well as in BLM‐induced PF, due to excessive collagen deposition (Dominguez et al., 2022; Lupher & Gallatin, 2006; Pilling et al., 2007; Qu et al., 2022; Rao et al., 2021). This excessive collagen deposition is attributable to increased fibroblast and myofibroblast activity as fibrotic lung diseases progress. Translationally, the increase in markers associated with this cellular activity is seen in human patients and in murine models of BLM‐induced PF (Ishida et al., 2023; Surolia et al., 2019). In our study, we used ELISA to evaluate protein levels of collagen I as a mature collagen marker, collagen III as a marker of new collagen deposition, and vimentin as a marker of fibroblast and myofibroblast activation. In our DB two‐hit experimental group, we observed no significant changes in collagen I content within lung tissue. We likely did not see substantial change in mature collagen content due to the conservative dosage of BLM and endpoint we chose for fibrotic development, as these two factors likely contributed to the slow progression of fibrosis. Using Fisher's Exact analysis of the ELISA data, we observed that the SB drug‐only control group and DB two‐hit experimental group were the only two groups with levels of collagen III above LOD. We believe these findings are suggestive of persistent active collagen deposition following lung injury. Interestingly, we did not observe significant changes in levels of vimentin; however, we did observe that the Exposure x Drug interaction main effect trended towards statistical significance. We hypothesize that the absence of significant changes in vimentin levels between groups may be indicative of the slow or moderate activation of fibroblasts and myofibroblasts following our lung injury regimen, but further investigation is warranted. We believe that these data imply slow fibrotic tissue remodeling and temporal changes in inflammation consistent with gradual progression of lung disease observed in humans. This supports the utilization of this two‐hit model schema for studying the cellular and molecular mechanisms underlying tissue damage and repair responses associated with lung disease development.

Following assessment of structural protein levels present within the lung following the two‐hit regimen, we investigated the presence of secreted cytokines in the airways associated with inflammatory processes. Previous studies have reported that cytokines associated with inflammatory processes, such as IL‐6, IL‐10, and TGFβ‐1 are present and significantly up‐regulated in the airways in a model of repetitive exposure to DE (Dominguez et al., 2022; Nordgren et al., 2015). It has also previously been shown that these same cytokines are significantly up‐regulated in the airways in dose‐dependent and endpoint‐dependent manners in models of BLM‐induced lung disease (Gul et al., 2023; Kadam & Schnitzer, 2024; Wynn & Ramalingam, 2012). In our novel two‐hit model, we observed no statistically significant changes in IL‐6, IL‐10, or total TGFβ‐1 protein levels in BALF in any of the experimental groups. We can conclude that while these proteins were present in all experimental groups, the two‐hit treatment regimen induced only mild production of these cytokines. Thus, these combined data may be indicative of a more balanced inflammatory environment within the lungs at the endpoint selected. Further investigation utilizing a broader array of cytokines associated with inflammation would provide a deeper understanding of the pulmonary inflammatory milieu and help better define the immunological landscape underlying these responses. Additionally, evaluation of oxidative stress markers in the two‐hit model compared to single models of inhaled dust exposure or BLM alone would provide insight into the contribution of these mechanisms to lung tissue damage and remodeling in response to heterogenous inhaled insults.

After characterizing the structural composition of the lungs following the two‐hit model regimen, we profiled the total and individual immune cell populations present. We have previously shown that repetitive exposure to DE leads to an increase in immune cell infiltration into the lungs, evident in BALF collected from study animals (Dominguez et al., 2022; Ulu et al., 2022; Warren et al., 2017). It has also been shown that BLM administration leads to an increase in airway immune cell infiltration (Gul et al., 2023; Kadam & Schnitzer, 2024). We aimed to recapitulate what has previously been shown in the literature by characterizing the total and differential immune cell counts in the BALF of animals from each model for comparison to the novel two‐hit model regimen. As expected, we observed total immune cell counts and differential cell counts in the BALF trending towards what has previously been established in the repetitive DE exposure model and the BLM only model (Barbayianni et al., 2018; Kadam & Schnitzer, 2024; Nordgren et al., 2015; Poole et al., 2009; Warren et al., 2017). It has not previously been established how a priming exposure, like organic dust, followed by a known driver of PF influences total or differential immune cell counts. However, we anticipated that the inflammatory environment within the lungs of DB two‐hit experimental animals would drive the cellular profile towards increased abundance of total cells, neutrophils, macrophages, and lymphocytes. Interestingly, we observed that the total immune cell counts, macrophages, and lymphocytes of the DB two‐hit experimental group were significantly influenced by the BLM drug treatment and not the DE exposure. While some neutrophilia was observed in this group, it was not statistically significant. Based on this cellular profile, we believe that the time allotted post final BLM administration allowed for mild resolution of the inflammation conferred by the repetitive DE exposure regimen (Warren et al., 2017). These data establish a foundation for more detailed characterization of cellular subtypes in this model, which could be further resolved using robust immunophenotyping approaches such as flow cytometry. Future investigations into the functional characteristics of the cells present in the BALF and lung homogenates would also provide context for understanding the balance between innate and adaptive immunity in this model.

This novel model generates robust physiological and mechanistic insights into the influence of heterogeneous insults on chronic lung disease development. Our study exhibits several strengths in justifying future use of this model. First, we developed this dual‐hit approach by implementing two well‐established and characterized murine models to synthesize a robust basis of knowledge in which the new findings could be compared. Secondly, we aimed to characterize the resultant lung injury of our dual‐hit model in a comprehensive manner using measures of animal weight, lung physiology, histopathology, structural cytokines, and inflammation at the cellular and molecular levels. Lastly, this two‐hit approach provides a practical way to bridge the existing gap in knowledge of how mechanisms initiated by serial inhalable toxicant exposures drive lung disease initiation and progression.

There were some limitations to this study that are important to note. First, the animals used for the two‐hit model ranged from 7 to 14 weeks in age, which could have introduced variability into the resultant physiological data and subsequent cytokine and histopathology data. Future studies that incorporate mice that fall within a shorter age range, such as 10 to 12 weeks, would likely lessen data variability from each group. Next, the sample size for these studies was small and resulted in limited statistical power, which likely affected the outcomes and conclusions that reached significance in the present assessment. A secondary limitation from the small sample size is that despite both male and female mice being utilized for the studies, we were statistically underpowered to assess for sex differences in all analyses performed. Additionally, we chose to utilize the established doses, routes of administration, and timelines of inhaled DE exposure and BLM to generate the dual‐hit model; however, we acknowledge that future studies that alter any one of these factors may greatly influence the amount of lung injury this model can generate. Finally, consideration of the temporal dynamics of inflammation and immune responses would strengthen the interpretation of the dual‐hit model and provide insight into optimizing the exposure regimen. Future studies incorporating time‐course analyses would resolve this limitation. Thus, our intent was to create a lung disease model that incorporates multiple heterogenous inhaled insults to provide a baseline of preliminary characterizations of resultant lung damage and repair responses that will inform the use of this model in future studies.

In conclusion, we developed a novel two‐hit model of inhaled occupational exposure‐induced lung disease to better interrogate the cellular and molecular mechanisms underlying the progression from persistent lung inflammation to fibrosis onset. We applied this model to determine the effects of serial inhalable toxicant exposure on animal health, as well as the resultant lung injury, and found that sequential heterogeneous exposures provide unique and translationally relevant insights for future mechanistic studies of early to mid‐stage fibrotic remodeling characteristic of sub‐chronic lung disease development.

## AUTHOR CONTRIBUTIONS

**Melea Barahona:** Conceptualization; data curation; formal analysis; investigation; methodology. **Ashley DeBie:** Investigation; methodology. **Logan S. Dean:** Investigation; methodology. **Bethany Klemp:** Investigation. **Kaylee Jones:** Methodology. **Emmanuel O. Oyewole:** Investigation. **Mäelis Wahl:** Investigation. **Morgan Pauly:** Investigation. **Casey McDermott:** Investigation. **Francisco J. Salguero:** Formal analysis. **G. Brooke Anderson:** Methodology; project administration; resources; supervision. **Marcela Henao‐Tamayo:** Methodology; project administration; resources; supervision. **Tara M. Nordgren:** Conceptualization; formal analysis; funding acquisition; investigation; methodology; project administration; resources; supervision.

## FUNDING INFORMATION

T.M.N., National Heart, Lung, and Blood Institute, R01HL158926. M.B., National Institute of Health, Quantitative Cell and Molecular Biology Training Program, T32GM132057.

## CONFLICT OF INTEREST STATEMENT

The authors certify that they have no financial, commercial, or personal relationships with third parties that could constitute a conflict of interest regarding the research conducted and reported herein.

## ETHICS STATEMENT

All mouse studies were conducted according to protocols reviewed and approved by the Colorado State University (CSU) Institutional Animal Care and Use Committee (Protocol Number 2887).

## Data Availability

The raw data supporting the findings of this article are available from the corresponding author upon request.
